# The Influence of Social Support on the Relationship between Emotional Demands and Health of Hospital Nurses: A Cross-Sectional Study †

**DOI:** 10.3390/healthcare9020115

**Published:** 2021-01-22

**Authors:** Hyoung Eun Chang, Sung-Hyun Cho

**Affiliations:** 1 College of Nursing, Konyang University, Daejeon 35365, Korea; 2 Research Institute of Nursing Science, College of Nursing, Seoul National University, Seoul 03080, Korea; sunghcho@snu.ac.kr

**Keywords:** emotional demands, social support, burnout, stress, nurse

## Abstract

Social support reduces the negative results of emotional labor. A more detailed analysis should be performed to facilitate adequate social support for nurses. Therefore, this study was conducted to examine the relationships among nurses’ emotional demands, social support, and health. A cross-sectional survey was conducted at two tertiary hospitals in South Korea. A sample of 117 nurses from eight units participated. Between-group differences in the main variables were analyzed using the *t*-test or Mann–Whitney test, and analysis of variance or the Kruskal–Wallis test. Nurses were classified into eight groups according to emotional demands and type of social support, and the effects of social support were analyzed based on mean scores. Greater social support from colleagues was associated with better health on all measures. However, greater social support from supervisors was associated with a higher incidence of burnout, stress, and sleeping troubles. Nurses’ high emotional demands must be managed actively by hospitals to maintain and promote their health. Providing appropriate social support with consideration of the nurse’s unit experience would help decrease the effects of emotional demands. Enhancing social support from nursing colleagues is a powerful way to manage the negative effects of nurses’ emotional demands.

## 1. Introduction

Nurses perform their jobs in a complex environment that requires various competencies, thereby causing considerable stress. Typically, nurses face physical demands to provide direct care, as well as psychosocial demands that arise while providing nursing care to patients. These demands combine to determine the workload of nurses. Previous studies have found that workload and intensity of work affect nurses’ burnout and turnover intention [1,2,3]. In addition, tension at work increases the duration of nurses’ absenteeism [4], and job demands have been shown to increase burnout in newly licensed nurses and negatively affect their mental health [5]. These results indicate that job demands are closely related both to job performance and to the health of the nurse performing the job.

Nursing is known to be a typical job with a high level of emotional labor [6,7]. According to June and Choi [8], the score of emotional demands of Korean office workers was lower than care workers in Denmark [9]. However, Korean nurses were reported to have scores more than 20 points higher [3] than Korean office workers [8]. High emotional demands have been found to be positively related to poor health, sleeping troubles, work-family conflict, stress [3], burnout, and intent to leave [3,10], and negatively related to job satisfaction and job performance of hospital nurses [3,10].

Previous studies based on the job demands-resources model have explained the relationships among job demands, job resources, and job performance [5,11]. According to this model, job demands have a negative impact on health through exhaustion, while job resources, which are motivated by workers’ enthusiasm, are related to positive job performance [12]. It was found that burnout was high when job demands such as physical workload, time pressure, work environment, and shift work were high, and enthusiasm was lowered when job resources such as compensation, feedback, participation, job stability, and support from one’s supervisor were low [13]. Additionally, in a previous study conducted in Korea, nurses with a high workload had higher burnout and lower job satisfaction [14].

In addition, the job demands-resources model emphasizes that nurses’ performance can be improved through job resources [12], which include organizational and social aspects for nurses working in hospitals. Opportunities for education or development, appropriate compensation, autonomy, work control, and job security are on the organizational side, while leadership, social support, and cooperation of one’s supervisor are on the social side [15]. These job resources increase job satisfaction and job performance [16,17] and reduce burnout due to job demands [15,18]. Several studies from South Korea (hereafter, Korea) have dealt with job resources related to senior nurses, especially leadership and social support from the supervisor [14,19] because the influence of senior nurses is considered to be especially important in Korea, reflecting its highly hierarchical organizational culture in nursing. Yom [14] showed that a higher workload was related to lower support from supervisors, and higher emotional depletion was related to higher dehumanization and lower self-achievement.

The results of previous research demonstrate that proper job demands and job resources are important in the nursing environment. According to a previous study, burnout takes place because an individual performing a job is more sensitive to job demands than to job resources [12]; therefore, maintaining proper job demands will be especially important. However, there is expected to be a difference in the roles and effects of low and high job resources depending on whether job demands are low or high.

Therefore, this study aimed to measure emotional demands, which are the main job demands of nurses, and to understand their effects on the health of nurses. In addition, this study investigated the influence of social support on the relationship between nurses’ emotional demands and health. Through this, we would like to suggest policies and programs to properly manage nurses’ job demands and job resources.

## 2. Materials and Methods

### 2.1. Study Design

This cross-sectional study was conducted to determine the relationships among emotional demands, social support, and health of registered nurses using survey data.

### 2.2. Setting and Participants

A sample of 117 registered nurses from eight nursing units at two tertiary university hospitals located in metropolitan cities in Korea were asked to participate in the survey. We targeted four types of nursing units (medical wards, surgical wards, intensive care units [ICUs], and emergency rooms [ERs]) to include units dedicated to the delivery of direct nursing care, with nurses working three shifts; therefore, special care units such as those in outpatient departments were excluded. The survey included all staff nurses working in the nursing units, except those who were excluded from evening and night shifts due to pregnancy. Among nurses who wanted to participate voluntarily in the four nursing units selected from each hospital, we tried to recruit about 15 nurses from each unit with a consistent ratio for each career stage. However, one of the nursing units selected for the study had a relatively small number of nurses who were able to work all three shifts and also had dropouts, so data from only 12 nurses from that ward were included in the analysis.

### 2.3. Data Collection

A researcher unaffiliated with the participating hospitals performed data collection. After obtaining permission from the nursing departments, we advertised the survey on the website of each hospital. After eight nursing units were selected at two tertiary university hospitals, we asked all nurses to participate in the survey voluntarily. Data were collected between 3 January and 15 March 2017.

### 2.4. Measures

Emotional demands, social support, and nurses’ health were measured using the second version of the Copenhagen Psychosocial Questionnaire (COPSOQ II) [9]. The Korean-language version of the COPSOQ II developed by June and Choi [8] was used in this study. The first version of the Copenhagen Psychosocial Questionnaire was developed in 2005 [20]; subsequently, the second version was developed in 2010 [9] and the tool has been validated in several studies [3,7,8,21,22,23].

Emotional demands were measured with four questions using a 5-point scale. The response options were as follows: (1) always, (2) often, (3) sometimes, (4) seldom, and (5) never/hardly ever. Social support was measured in terms of two types of support (from supervisors and from colleagues), each of which was evaluated using three questions with a 5-point scale. The response options were as follows: (1) always, (2) often, (3) sometimes, (4) seldom, and (5) never/hardly ever. Nurses’ health was measured with four sub-scales (burnout, stress, depressive symptoms, and sleeping troubles), each of which consisted of four items with a 5-point scale. The response options were as follows: (1) all the time, (2) a large part of the time, (3) part of the time, (4) a small part of the time, and (5) not at all.

The Cronbach alpha values of emotional demands, social support from colleagues, social support from supervisors, burnout, stress, depressive symptoms and sleeping troubles were 0.70, 0.70, 0.74, 0.82, 0.80, 0.78, and 0.88, respectively.

### 2.5. Data Analysis

The items measuring the dimensions with five response categories were scored as 0, 25, 50, 75, and 100. They were calculated as the mean of the item responses, with higher scores indicating feelings of more intense emotional demands, social support, burnout, stress, depressive symptoms, and sleeping troubles.

Differences between emotional demands, social support, and health according to the general characteristics of the nurses were analyzed using the t-test or Mann–Whitney test, and analysis of variance or the Kruskal–Wallis test. When there were significant differences between groups, a multiple-comparison post hoc test was conducted using the Bonferroni correction.

In order to analyze the effects of social support on the relationship between emotional demands and health, nurses were classified into four groups according to emotional demands/social support from supervisors, and four groups according to emotional demands/social support from colleagues. Specifically, the groups were categorized as low emotional demands and high social support (low-high), low emotional demands and low social support (low-low), high emotional demands and high social support (high-high), and high emotional demands and low social support (high-low). Therefore, groups for support from supervisors were categorized into four groups: Low-High, Low-Low, High-High, and High-Low (hereafter referred to as the LHs, LLs, HHs, and HLs). Groups for support from colleagues were also categorized into four groups: Low-High, Low-Low, High-High, and High-Low (hereafter referred to as and the LHc, LLc, HHc, and HLc). Theoretically, the most favorable group would be LH, followed in order by LL, HH, and HL. To classify emotional demands and social support as high and low, the median scores of emotional demands (75.0), social support from supervisors (66.7), and social support from colleagues (66.7) were used. To be more specific, for example, the LHs group consisted of nurses who perceived their emotional demands to be lower than 75.0 points and their social support from supervisors to be higher than 66.7 points.

### 2.6. Ethical Considerations

The study received institutional review board approval (IRB No. 1701/001-002) from Seoul National University. Nurses participated in the study on a voluntary basis and completed a written consent form prior to answering the survey questionnaires. They were informed that they could discontinue participation in the survey at any time without any harmful consequences. Their personal information was managed in a master file separately from the datasets used for analysis.

## 3. Results

Most of the nurses who participated in the study were female (94.0%) and unmarried (93.1%). The average age was 26.2 years old and the majority of them had a bachelor’s degree (83.8%). Their average duration of unit experience was 2.9 years. Nurses working in medical wards accounted for 23.1% (*n* = 27) of the participants (*N* = 117), and nurses from surgical wards, ICUs, and ERs accounted for the same proportion (*n* = 30, 25.6%).

### 3.1. Comparison of the Main Variables According to Nurses’ General Characteristics

Table 1 presents the means and standard deviations of emotional demands, social support from supervisors, social support from colleagues, burnout, stress, depressive symptoms, and sleeping troubles. The mean score of emotional demands was 62.0 ± 15.5. The mean score of social support from colleagues (66.2 ± 14.2) was higher than that of social support from supervisors (60.7 ± 16.3). The mean score of burnout (68.3 ± 14.6) was highest, followed by stress (65.4 ± 15.7), sleeping troubles (53.5 ± 20.0), and depressive symptoms (51.8 ± 17.7).

The emotional demands of nurses were the highest in medical wards (69.4 ± 11.8), followed by surgical wards (65.6 ± 16.6), ERs (59.5 ± 17.0), and ICUs (54.0 ± 11.4), with a significant difference (*p* = 0.001). Older age and an associate’s degree were non-significantly associated with higher scores for emotional demands. A longer duration of unit experience was associated with a significantly higher score for emotional demands (67.9 ± 15.9, *p* = 0.003). Social support from supervisors showed no significant differences according to the nursing unit but was significantly higher in younger participants (65.6 ± 13.7, *p* = 0.005). Nurses with associate’s degrees experienced higher support from supervisors (63.2 ± 15.3), but this was non-significant, and nurses with shorter duration of unit experience experienced significantly higher support from supervisors (66.4 ± 15.4, *p* = 0.001). Social support from colleagues also showed no significant differences across nursing units or according to age, unlike support from supervisors. Nurses with a bachelor’s or higher degree experienced higher social support from colleagues (66.9 ± 14.1), and nurses with 1–3 years of unit experience showed highest social support from colleagues (68.6 ± 13.5), but these differences were not significant. Burnout, stress, and depressive symptoms were highest among nurses working in medical wards, followed by those working in surgical wards, ERs, and ICUs, and the difference was statistically significant. No significant differences were found in burnout and depressive symptoms according to age, education level, and unit experience. Stress was not significantly different according to age and unit experience, but nurses with an associate’s degree showed significantly higher stress (72.4 ± 12.5, *p* = 0.042). There were no significant differences in sleeping troubles according to nursing unit, age, education level, and unit experience.

### 3.2. Emotional Demands and Social Support According to Nurses’ General Characteristics

Table 2 presents the distribution of the LHs, LLs, HHs, and HLs groups with reference to emotional demands and social support from supervisors according to nurses’ general characteristics. There were 17, 33, 18, and 49 nurses in the LHs, LLs, HHs, and HLs groups, respectively. Medical ward nurses tended to have high emotional demands and low social support from supervisors (*n* = 18, 36.7%) and surgical ward nurses generally experienced high emotional demands. ICU nurses and ER nurses tended to have low social support from supervisors. Nurses under 28 years of age generally experienced high social support from supervisors. In all groups, more than 80% had bachelor’s degree or more, and the HLs group had the highest proportion of nurses with associate’s degrees (*n* = 8). Nurses with longer unit experience generally had lower scores of social support from supervisors.

Table 2 also presents the distribution of these four groups classified in terms of emotional demands and social support from colleagues according to the nurses’ general characteristics. The LHc, LLc, HHc, and HLc groups contained 18, 32, 28, and 39 nurses, respectively. ICU nurses (*n* = 10, 55.6%) were the most common in the LHc group, medical ward nurses predominated in the LLc group (*n* = 10, 31.3%), ER nurses were most common in the HHc group (*n* = 9, 32.1%), and medical ward nurses predominated in the HLc group (*n* = 15, 38.5%). Medical ward nurses mostly had high emotional demands, surgical ward nurses tended to have low social support from colleagues. ICU nurses tended to have low emotional demands, and ER nurses generally had high emotional demands and low social support from colleagues.

Younger nurses tended to have higher emotional demands and lower social support from colleagues. In all groups, more than 80% had a bachelor’s degree or more and nurses with an associate’s degree predominated in the HL group (*n* = 7) and were under-represented in the HH group (*n* = 4). Generally, nurses with longer unit experience experienced higher emotional demands.

### 3.3. Relationships between Nurses’ Health and Emotional Demands/Social Support

**Figure 1:**
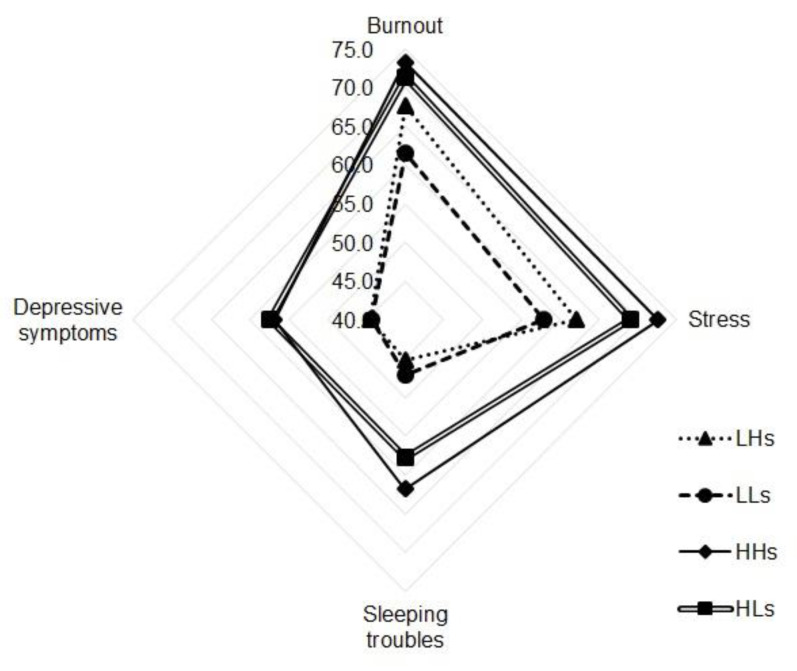
Nurses’ health in four groups defined by emotional demands/social support from supervisors. Note: LHs = low emotional demands/high social support from supervisors, LLs = low emotional demands/low social support from supervisors, HHs = high emotional demands/low social support from supervisors, HLs = high emotional demands/low social support from supervisors.

**Figure 2:**
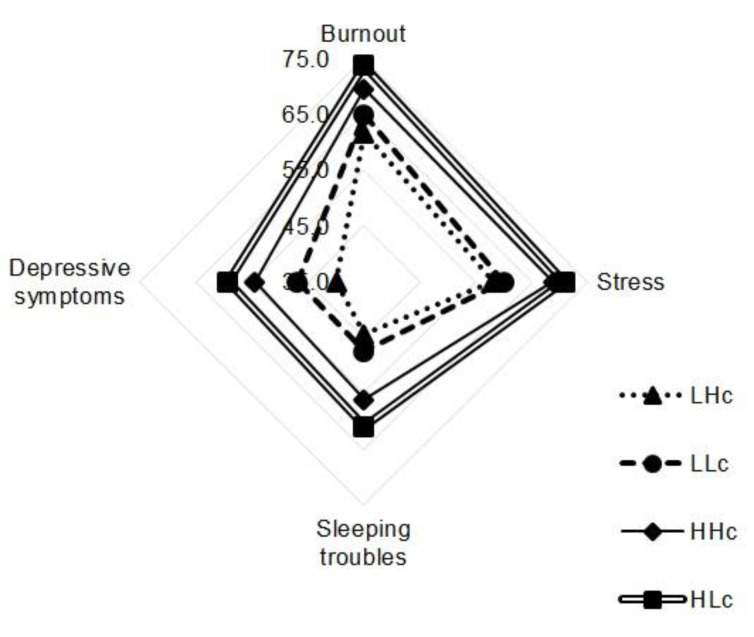
Nurses’ health in four groups defined by emotional demands/social support from colleagues. Note: LHc = low emotional demands/high social support from colleagues, LLc = low emotional demands/low social support from colleagues, HHc = high emotional demands/low social support from colleagues, HLc = high emotional demands/low social support from colleagues.

Table 3 and Figure 1 and Figure 2 present comparisons of the differences in health results among the eight groups classified by emotional demands and social support from supervisors or colleagues.

First, for the groups classified by emotional demands and social support from supervisors, burnout was highest in the HHs group (73.3 ± 14.0), followed by the HLs (71.3 ± 14.0), LHs (67.6 ± 11.9), and LLs (61.6 ± 15.2) groups. The difference among groups was statistically significant (*p* = 0.006), especially between the LLs and HHs (*p* = 0.024) groups and between the LLs and HLs (*p* = 0.012) groups, which showed significant difference through multiple comparisons. Stress was highest in the HHs group (72.6 ± 16.1), followed by the HLs (69.0 ± 13.7), LHs (62.1 ± 14.1), and LLs (57.8 ± 16.1) groups. The difference among groups was significant (*p* = 0.002), and significance was verified by multiple comparisons between the LLs and HHs groups (*p* = 0.005) and between the LLs and HLs groups (*p* = 0.006). Depressive symptoms were highest in the HLs group (57.4 ± 14.1), followed by the HHs (56.9 ± 20.1), LHs (44.5 ± 14.3), and LLs (44.3 ± 19.1) groups, with statistically significant differences among all four groups (*p* = 0.003) and between the LHs and HLs groups (*p* = 0.035), the LLs and HHs groups (*p* = 0.052), and the LLs and HLs groups (*p* = 0.004). Sleeping troubles were highest in the HHs group (61.8 ± 20.9), followed in order by the HLs (57.8 ± 20.3), LLs (47.0 ± 16.5), and LHs (45.2 ± 19.3) groups. The difference among groups was significant (*p* = 0.004), especially between the LLs and HHs (*p* = 0.047) groups, as proved by multiple comparisons.

As such, the groups with more support from supervisors did not show better health results, as was expected on theoretical grounds. In particular, for pairs of groups differing only in terms of social support from supervisors, burnout, stress, and depression were found to be higher in groups with higher social support from supervisors (Figure 1).

Second, for the four groups classified by emotional demands and social support from colleagues, burnout was highest in the HLc group (73.6 ± 13.9), followed by the HHc (69.4 ± 13.9), LLc (64.8 ± 13.1), and LHc (61.5 ± 16.6) groups. The difference was statistically significant (*p* = 0.009), and the difference between the LHc and HLc groups (*p* = 0.017) showed significance through multiple comparisons. Stress was highest in the HLc group (70.8 ± 14.4), followed by the HHc (68.8 ± 14.5), LLc (60.0 ± 16.2), and LHc (58.0 ± 14.3) groups, and the difference was significant (*p* = 0.002). Multiple comparison testing showed significant differences between the LHc and HLc groups (*p* = 0.016) and the LLc and HLc groups (*p* = 0.015). Depression was highest in the HLc group (59.3 ± 15.2), followed by the HHc (54.5 ± 16.4), LLc (46.9 ± 17.5), and LHc (39.9 ± 16.9) groups, and the difference was significant (*p* < 0.001). The LHc and HHc groups (*p* = 0.025), the LHc and HLc groups (*p* < 0.001), and the LLc and HLc groups (*p* = 0.012) showed significant differences according to multiple comparisons. Sleeping troubles were highest in the HLc group (60.9 ± 20.3), followed by the HHc (56.0 ± 20.5), LLc (47.5 ± 18.0), and LHc (44.4 ± 16.5) groups, with statistical significance (*p* = 0.003). The LHc and HLc groups (*p* = 0.016) and the LLc and HLc groups (*p* = 0.020) showed significance in multiple comparisons.

Nurses’ health was better in groups with lower emotional demands and higher social support, as was expected on theoretical grounds (Figure 2).

## 4. Discussion

This study investigated the effects of emotional demands and social support in nurses’ work environment on their health.

Differences were found in emotional demands, burnout, stress, and depressive symptoms across nursing units. The emotional demands of nurses working in general wards were highest and the emotional demands of ICU nurses were lowest, whereas Cho et al. [3] found that the emotional demands of ICU nurses were higher than those of general ward nurses, and that the emotional demands of nurses in oncology patient wards were higher than those of ICU nurses. The results of our study and Cho et al. [3] can be compared because emotional demands were investigated with the same tool, and their result partially supports the finding of our study that the highest emotional demands were found among nurses working in internal medicine wards. In this study, emotional demands, burnout, stress, and depressive symptoms were all highest in the internal medicine wards, followed in order by the surgical ward, ERs, and ICUs, with statistically significant differences. In previous studies, burnout [24] and stress [25] were found to be closely related to work intensity and emotional burnout. Thus, emotional demands and health outcomes should show the same pattern of correlation. Moreover, Yoon and Kim [26] found that nurses in internal medicine wards had the highest degree of depression, whereas nurses in special wards had slightly higher depression than those in surgical wards. Further research should explore differences in the work content and unit characteristics that increase nurses’ psychological burden and investigate how these intensify work. In addition, emotional demands were found to be higher as unit experience increased. As nurses become more accustomed to work, the burden of interpersonal relationships and job responsibilities is expected exceed the psychological burden of the workload. Since emotional demands were found to be an important factor affecting nurses’ burnout, stress, depression, and sleep quality [3], additional research on work content and work environment in relation to emotional demands is needed. It will also be necessary to identify the cause of increasing emotional demands in relationships with senior nurses or interpersonal relationships with people including colleague nurses, physicians, patients, and patients’ families, and to plan specific interventions accordingly.

In this study, nurses perceived higher social support from colleagues and supervisors than white-collar workers in a previous study [8]. The forms of social support investigated herein may be more closely related to aspects of human relationships or leadership than job characteristics. Social support from colleagues did not show any significant differences according to the general characteristics of nurses, but older nurses and those with longer unit experienced perceived less social support from supervisors. This may be because more experienced nurses have relatively fewer nurses who are senior to them, as well as because as career nurses gain experience, their role may shift from being supported by senior nurses to providing support for junior nurses. In addition, greater social support from colleagues had positive effects on health outcomes. However, social support from supervisors showed the opposite relationship. For example, poorer outcomes were found for burnout, stress, and sleeping troubles in groups with high social support from supervisors, regardless of their emotional demands. Supervisors’ interest and support might not have directly led to help and stress relief, whereas colleagues immediately relieve the work burden or provide more effective emotional support. In addition, when nurses are asked about their work and any problems that they have encountered, their reactions may depend on who asks the question. That is, it can be difficult to express difficulties and ask for help from one’s direct supervisor. It is also noteworthy that the eight groups defined by levels of emotional demands and social support did not have an even distribution. For example, ICU nurses tended to have low social support and nurses in surgical wards generally faced high emotional demands, and ICU nurses had the lowest levels of burnout, stress, and sleeping troubles. This result reflects the previously reported finding that individuals are likely to be more sensitive to job demands than to job resources [12]. In other words, it seems that social support from supervisors (a job resource) does not sufficiently counteract the negative health and well-being caused by high job demands.

However, in this study, social support from colleagues had positive effects between emotional demands and nurses’ health. In other words, increased social support from colleagues in an environment with the same emotional demands effectively reduces burnout, stress, depressive symptoms, and sleeping troubles. Nurses have an irregular life due to shift work, making it difficult to engage in hobbies or spend time with family and friends. Therefore, support from colleague nurses may be especially important. If hospitals provided nurses opportunities to socialize outside working hours, their relationships would be strengthened, and stress would be relieved.

Nurses who were older and with longer unit experience are thought to have had relatively low social support from supervisors because of their professional skill. However, since their emotional demands were relatively high, social support suitable for their role is needed. It may be helpful for them to regularly consult with supervisors, participate in programs to reduce their stress, or take part in educational programs that strengthen communication skills in interpersonal relationships. Developing protocols by standardizing interpersonal communication methods appropriate to the characteristics of nursing units will also help reduce occupational burdens at the personal level.

Another issue to be pointed out is that social support (a job resource) can change in response to changes in the medical environment. As the current coronavirus disease 2019 (COVID-19) pandemic is having unprecedented impacts throughout the world, the medical environment is in a crisis situation, and it is necessary to discuss the social support that medical professionals need and can actually be provided. Especially in a pandemic situation, nurses have to take care of sudden increase in patients and accordingly face a heavy workload. Kim, Lee, and Cho [27] conducted a study that reflects this situation at present and revealed that nurses caring for COVID-19 patients had low job retention intention, and encouragement and support through mass media and national encouragement were effective ways to reduce the turnover of nurses caring for COVID-19 patients. The scope of our study was limited to social support from colleagues and supervisors; however, it was found that nurses’ social support expanded in a national disaster situation [27]. Therefore, research on the job demands and job resources of nurses reflecting social changes and changes in the medical environment should be carried out in further studies. On the basis of such research, it is expected that the resources needed by nurses will be provided, and the quality of nursing and patient safety will be improved.

This study has several limitations. First, the cross-sectional study design precludes the inference of causal relationships. Second, this study was conducted at tertiary university hospitals in metropolitan cities, and the nurses who participated in the survey were relatively younger. Moreover, rather few subjects from two institutions in Korea were analyzed; therefore, especially given the short survey that was used, our findings may not be readily generalizable. Third, relationships of overall health to aspects of daily life outside of work were not investigated.

## 5. Conclusions

Our study results highlight the importance of managing emotional demands and providing adequate social support for nurses. Nurses’ high emotional demands must be managed actively by hospitals to maintain and promote their health, and interventions to provide social support of nurses will both affect their job performance and health.

The emotional demands of nurses varied according to the nursing unit, nurses’ age, and nurses’ unit experience. Based on these results, it will be possible to plan and implement appropriate interventions for nurses according to their unit or individual characteristics. Providing appropriate social support with consideration of nurses’ unit experience would be helpful for decreasing the effects of emotional demands. Additionally, by enhancing social support from nursing colleagues, the negative effects of nurses’ emotional demands could be diminished. However, care should be taken to provide social support from supervisors in an effective manner.

## Figures and Tables

**Table 1:** Comparison of the main variables according to general characteristics (*N* = 117).

Characteristics/Categories	Emotional Demands	Social Support from Supervisors	Social Support from Colleagues	Burnout	Stress	Depressive Symptoms	Sleeping Troubles
	M ± SD						
**Overall**	62.0 ± 15.5	60.7 ± 16.3	66.2 ± 14.2	68.3 ± 14.6	65.4 ± 15.7	51.8 ± 17.7	53.5 ± 20.0
**Unit type**							
Medical ward ^a^	69.4 ± 11.8	60.8 ± 18.6	63.9 ± 15.2	74.5 ± 12.8	70.1 ± 12.7	59.5 ± 11.9	55.3 ± 18.4
Surgical ward ^b^	65.6 ± 16.6	64.2 ± 14.7	64.7 ± 15.6	69.6 ± 13.7	67.1 ± 16.7	53.5 ± 20.3	57.9 ± 23.8
Intensive care unit ^c^	54.0 ± 11.4	60.8 ± 15.5	70.0 ± 12.5	60.6 ± 17.0	58.8 ± 16.0	44.4 ± 18.1	47.3 ± 19.5
Emergency room ^d^	59.5 ± 17.0	56.9 ± 15.2	65.8 ± 13.2	69.2 ± 11.1	66.0 ± 15.5	50.4 ± 16.2	53.8 ± 17.0
F/H/t/U (*p*)	16.4 (0.001) ^†^	3.2 (0.364) ^†^	4.2 (0.239) ^†^	12.3 (0.006) ^†^	10.5 (0.015) ^†^	10.8 (0.013) ^†^	6.5 (0.092) ^†^
Multiple comparison	a,b > c			a > c	a > c	a > c	
**Age (years)**							
22–24 ^a^	59.7 ± 13.0	65.6 ± 13.7	67.4 ± 12.8	71.3 ± 12.1	67.8 ± 13.8	53.2 ± 16.5	51.0 ± 21.2
25–27 ^b^	61.3 ± 18.7	61.5 ± 16.7	68.3 ± 13.6	66.4 ± 15.5	62.7 ± 19.1	49.4 ± 21.2	53.6 ± 19.5
≥28 ^c^	66.0 ± 13.8	52.9 ± 16.5	61.7 ± 16.0	66.6 ± 16.4	65.4 ± 13.3	52.7 ± 14.4	57.0 ± 19.0
F/H/t/U (*p*)	2.9 (0.233) ^†^	10.6 (0.005) ^†^	3.4 (0.185) ^†^	2.7 (0.257) ^†^	2.2 (0.336) ^†^	0.6 (0.574)	0.9 (0.429)
Multiple comparison		a > c					
**Education**							
Associate’s degree ^a^	67.6 ± 17.1	63.2 ± 15.3	61.8 ± 13.8	71.7 ± 10.4	72.4 ± 12.5	58.8 ± 15.8	57.4 ± 19.7
Bachelor’s degree or higher ^b^	61.0 ± 15.1	60.3 ± 16.4	66.9 ± 14.1	67.8 ± 15.2	64.2 ± 15.9	50.6 ± 17.7	52.9 ± 20.1
F/H/t/U (*p*)	1196.5 (0.135) ^††^	1061.0 (0.653) ^††^	762.0 (0.060) ^††^	1142.5 (0.279) ^††^	1267.0 (0.042) ^††^	1.8 (0.074)	1132.0 (0.319) ^††^
Multiple comparison					a > b		
**Duration of unit experience (year)**							
<1 ^a^	55.7 ± 15.3	66.4 ± 15.4	67.8 ± 13.3	70.9 ± 13.7	67.2 ± 14.5	53.5 ± 17.6	50.8 ± 21.2
1–2 ^b^	62.2 ± 13.4	62.6 ± 14.6	68.6 ± 13.5	67.6 ± 13.2	64.4 ± 15.7	50.4 ± 17.6	53.5 ± 18.9
≥3 ^c^	67.9 ± 15.9	52.7 ± 16.2	61.7 ± 15.0	66.6 ± 16.9	64.7 ± 17.1	51.5 ± 18.1	56.3 ± 20.3
F/H/t/U (*p*)	6.2 (0.003)	13.8 (0.001) ^†^	5.0 (0.084) ^†^	1.1 (0.577) ^†^	0.6 (0.751) ^†^	0.3 (0.734)	2.2 (0.332) ^†^
Multiple comparison	a < c	a > b,c					

Note: M = mean, SD = standard deviation, ^†^ Kruskal–Wallis test, ^††^ Mann–Whitney Test.

**Table 2:** Distribution of eight groups (defined by emotional demands/social support from supervisors and emotional demands/social support from colleagues) according to general characteristics (*N* = 117).

Characteristics/Categories	Emotional Demands/Social Support from Supervisors					Emotional Demands/Social Support from Colleagues				
	LHs (*n* = 17)	LLs (*n* = 33)	HHs (*n* = 18)	HLs (*n* = 49)	Total	LHc (*n* = 18)	LLc (*n* = 32)	HHc (*n* = 28)	HLc (*n* = 39)	Total
	*n*(%)					*n*(%)				
Unit type										
Medical ward	2 (11.8)	3 (9.1)	4 (22.2)	18 (36.7)	27 (23.1)	1 (5.6)	4 (12.5)	7 (25.0)	15 (38.5)	27 (23.1)
Surgical ward	5 (29.4)	8 (24.2)	7 (38.9)	10 (20.4)	30 (25.6)	3 (16.7)	10 (31.1)	7 (25.0)	10 (25.6)	30 (25.6)
Intensive care unit	7 (41.2)	12 (36.4)	4 (22.2)	7 (14.3)	30 (25.6)	10 (55.6)	9 (28.1)	5 (17.9)	6 (15.4)	30 (25.6)
Emergency room	3 (17.7)	10 (30.3)	3 (16.7)	14 (28.6)	30 (25.6)	4 (22.2)	9 (28.1)	9 (32.1)	8 (20.5)	30 (25.6)
Age (years)										
22–24	12 (70.6)	9 (27.3)	7 (38.9)	17 (34.7)	45 (38.5)	10 (55.6)	11 (34.4)	10 (35.7)	14 (35.9)	45 (38.5)
25–27	4 (23.5)	13 (39.4)	7 (38.9)	16 (32.7)	40 (34.2)	6 (33.3)	11 (34.4)	10 (35.7)	13 (33.3)	40 (34.2)
≥28	1 (5.9)	11 (33.3)	4 (22.2)	16 (32.7)	32 (27.4)	2 (11.1)	10 (31.3)	8 (28.6)	12 (30.8)	32 (27.4)
Education										
Associate’s degree	3 (17.7)	3 (9.1)	3 (16.7)	8 (16.3)	17 (14.5)	0 (0)	6 (18.8)	4 (14.3)	7 (18.0)	17 (14.5)
Bachelor’s degree or higher	14 (82.4)	30 (90.9)	15 (83.3)	41 (83.7)	100 (85.5)	18 (100.0)	26 (81.3)	24 (85.7)	32 (82.1)	100 (85.5)
Duration of unit experience (years)										
<1	12 (70.6)	11 (33.3)	6 (33.3)	8 (16.3)	37 (31.6)	11 (61.1)	12 (37.5)	5 (17.9)	9 (23.1)	37 (31.6)
1–2	4 (23.5)	12 (36.4)	8 (44.4)	19 (38.8)	43 (36.8)	5 (27.8)	11 (34.4)	14 (50.0)	13 (33.3)	43 (36.8)
≥3	1 (5.9)	10 (30.3)	4 (22.2)	22 (31.6)	37 (31.6)	2 (11.1)	9 (28.1)	9 (32.1)	17 (43.6)	37 (31.6)

Note: The LHs, LLs, HHs, and HLs groups were categorized by the level (high vs. low) of emotional demands and social support from supervisors. The LHc, LLc, HHc, and HLc groups were categorized by the level (high vs. low) of emotional demands and social support from colleagues.

**Table 3:** Nurses’ perceived health in eight groups defined by emotional demands/social support from supervisors and emotional demands/social support from colleagues (*N* = 117).

Group	Emotional Demands/Social Support from Supervisors					Emotional Demands/Social Support from Colleagues			
	Burnout	Stress	Depressive Symptoms	Sleeping Troubles	Group	Burnout	Stress	Depressive Symptoms	Sleeping Troubles
	M ± SD					M ± SD			
LHs ^a^	67.6 ± 11.9	62.1 ± 14.1	44.5 ± 14.3	45.2 ± 19.3	LHc ^a^	61.5 ± 16.6	58.0 ± 14.3	39.9 ± 16.9	44.4 ± 16.5
LLs ^b^	61.6 ± 15.2	57.8 ± 16.1	44.3 ± 19.1	47.0 ± 16.5	LLc ^b^	64.8 ± 13.1	60.0 ± 16.2	46.9 ± 17.5	47.5 ± 18.0
HHs ^c^	73.3 ± 14.0	72.6 ± 16.1	56.9 ± 20.1	61.8 ± 20.9	HHc ^c^	69.4 ± 13.9	68.8 ± 14.5	54.5 ± 16.4	56.0 ± 20.5
HLs ^d^	71.3 ± 14.0	69.0 ± 13.7	57.4 ± 14.1	57.8 ± 20.3	HLc ^d^	73.6 ± 13.9	70.8 ± 14.4	59.3 ± 15.2	60.9 ± 20.3
F/H (*p*)	12.5 (0.006) ^†^	14.6 (0.002) ^†^	13.7 (0.003) ^†^	13.3 (0.004) ^†^	F/H (*p*)	11.5 (0.009) ^†^	14.5 (0.002) ^†^	7.1 (<0.001)	14.0 (0.003) ^†^
Multiple comparison	b < c,d	b < c,d	a,b < d, b < c	b < c	Multiple comparison	a < d	a,b < d	a,b < d, a < c	a,b < d

Note: ^†^ Kruskal–Wallis test. The LHs, LLs, HHs, and HLs groups were categorized by the level (high vs. low) of emotional demands and social support from supervisors. The LHc, LLc, HHc, and HLc groups were categorized by the level (high vs. low) of emotional demands and social support from colleagues.

## Data Availability

The data presented in this study are available on request from the corresponding author. The data are not publicly available due to privacy or ethical restrictions.
